# RNA toxicity in non‐coding repeat expansion disorders

**DOI:** 10.15252/embj.2018101112

**Published:** 2019-11-13

**Authors:** Bart Swinnen, Wim Robberecht, Ludo Van Den Bosch

**Affiliations:** ^1^ Department of Neurosciences Experimental Neurology Leuven Brain Institute (LBI) KU Leuven – University of Leuven Leuven Belgium; ^2^ Laboratory of Neurobiology VIB, Center for Brain & Disease Research Leuven Belgium; ^3^ Department of Neurology University Hospitals Leuven Leuven Belgium

**Keywords:** C9ORF72 ALS/FTD, non‐coding repeat expansion disorders, RNA toxicity

## Abstract

Several neurodegenerative disorders like amyotrophic lateral sclerosis (ALS) and spinocerebellar ataxia (SCA) are caused by non‐coding nucleotide repeat expansions. Different pathogenic mechanisms may underlie these non‐coding repeat expansion disorders. While gain‐of‐function mechanisms, such as toxicity associated with expression of repeat RNA or toxicity associated with repeat‐associated non‐ATG (RAN) products, are most frequently connected with these disorders, loss‐of‐function mechanisms have also been implicated. We review the different pathways that have been linked to non‐coding repeat expansion disorders such as C9ORF72‐linked ALS/frontotemporal dementia (FTD), myotonic dystrophy, fragile X tremor/ataxia syndrome (FXTAS), SCA, and Huntington's disease‐like 2. We discuss modes of RNA toxicity focusing on the identity and the interacting partners of the toxic RNA species. Using the C9ORF72 ALS/FTD paradigm, we further explore the efforts and different methods used to disentangle RNA vs. RAN toxicity. Overall, we conclude that there is ample evidence for a role of RNA toxicity in non‐coding repeat expansion diseases.

## Graphical abstract

This review comprehensively delineates pathogenic mechanisms underlying various neurodegenerative disorders with special focus on disentangling toxicity associated with RNA species or RAN (repeat‐associated non‐ATG) products.

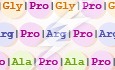

**Glossary**
ALS: amyotrophic lateral sclerosis
ASO: antisense oligonucleotide
DPR: dipeptide repeat protein
ELISA: enzyme‐linked immunosorbent assay
FTD: frontotemporal dementia
FTLD: frontotemporal lobe degeneration
FXTAS: fragile X tremor ataxia syndrome
HDL‐2: Huntington's disease‐like 2
hnRNP: heterogeneous nuclear ribonucleoprotein
HRE: hexanucleotide repeat expansion
iMNs: induced motor neurons
mRNP: messenger ribonucleoprotein
PET: positron emission tomography
RAN: repeat‐associated non‐ATG
RBP: RNA‐binding protein
rRBP: repeat RNA‐binding protein
SCA: spinocerebellar ataxia
UPS: ubiquitin‐proteasome system
UTR: untranslated region

## Introduction

### Non‐coding repeat expansion disorders

Several neurodegenerative disorders are caused by a non‐coding repeat expansion and are referred to as “non‐coding repeat expansion disorders”. So far, ten non‐coding repeat expansion disorders have been described (Table 1). Most of them are adult‐onset disorders and are phenotypically characterized by variable syndromes that include ataxia, cognitive dysfunction, motor neuron symptoms, and extra‐neuronal involvement (Table 1). The most frequent clinical syndromes are myotonic dystrophy, amyotrophic lateral sclerosis (ALS)/frontotemporal dementia (FTD), and spinocerebellar ataxia (SCA; Table 1). The repeat expansion can be located in the promoter region, the 5′UTR (untranslated region), an intron, an alternate exon, or the 3′UTR of the respective gene (Table 1). The repeat sequence is variable, ranging from trinucleotide to hexanucleotide repeats, and is in general characterized by a high GC content (except SCA10 and SCA31). The range of the repeat length in healthy individuals normally does not exceed 30 repeats (Table 1). However, the range of the (unambiguously) pathogenic repeat lengths is highly variable and can be subdivided into three classes. First, repeats in SCA12 and Huntington's disease‐like 2 (HDL‐2) are usually not longer than 100 repeats (Margolis *et* *al,* 2004; Dong *et* *al,* 2015). Second, repeats generally do not exceed a few hundred (± 200) in fragile X tremor ataxia syndrome (FXTAS; O'Donnell & Warren, 2002) although longer repeats are associated with fragile X syndrome due to *FMR1* loss of function (Verkerk *et* *al,* 1991). Third, repeats in all other diseases are mostly in the range of many hundreds up to a few thousands (Table 1). A clear length–phenotype correlation (i.e., more aggressive phenotype with increasing repeat length) has only been described in myotonic dystrophy types 1 and 2 (Udd & Krahe, 2012).

**Table 1:**
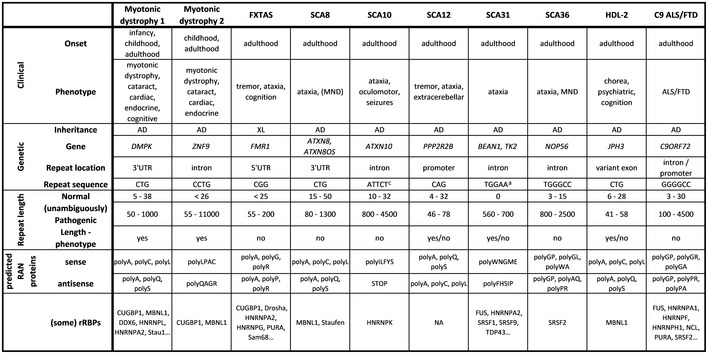
Non‐coding repeat expansion disorders

Overview of key features of all non‐coding repeat expansion disorders. Clinical features include age at onset (i.e., the main life phase(s)) and phenotypic presentation(s). Genetic features include inheritance pattern, gene containing the repeat expansion, location of the repeat in the respective gene, and sequence of the repeat. Data regarding repeat length include repeat length in healthy individuals, unambiguously pathogenic repeat lengths, and correlation between repeat length and phenotype. For each disease, all theoretical RAN proteins are described, both in sense and in antisense direction. Regarding possible mechanisms, rRBPs implicated in the disease are listed.

*Abbreviations*: AD, autosomal dominant; FXTAS, fragile X tremor ataxia syndrome; HDL, Huntington disease‐like; MND, motor neuron degeneration; rRBPs, repeat RNA‐binding proteins; SCA, spinocerebellar ataxia; XL, X‐linked.

Complex pentanucleotide (TAGAA, TAAAA, TAAAATAGAA).

“alteration” of function.

Impurity of repeat (associated with seizures).

### Three possible mechanisms

**Figure 1:**
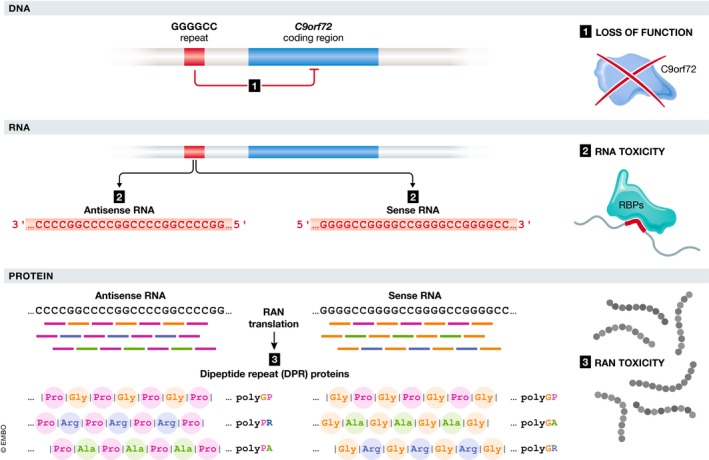
Three possible pathogenic mechanisms of non‐coding repeat expansion disorders—example given for C9ORF72 ALS/FTD First, the repeat expansion might interfere with the normal transcription of the C9ORF72 gene, leading to loss of function of the C9orf72 protein. Second, repeat‐containing mRNAs might bind to various RNA‐binding proteins, hence disturbing their normal function. This is called “RNA toxicity”. Third, the repeat RNA itself might unconventionally be translated into peculiar toxic RAN peptides. This is called “RAN toxicity”.

Three non‐mutually exclusive mechanisms have been linked to the pathogenesis of non‐coding repeat expansion disorders (Fig 1; example is given for C9ORF72 ALS/FTD (C9 ALS/FTD)). Repeat RNA can cause toxicity by directly interacting with repeat RNA‐binding proteins and thereby compromising their normal function (Miller *et* *al,* 2000). This is referred to as “RNA toxicity”. The repeat RNA might also induce toxicity indirectly by being translated into toxic proteins (Zu *et* *al,* 2011). This translation occurs in a non‐ATG‐dependent way called “repeat‐associated non‐ATG” (RAN) translation and generates RAN proteins (Zu *et* *al,* 2011). This type of toxicity is called “RAN toxicity”. Depending on the repeat type (i.e., tri‐, penta‐, or hexanucleotide) and the specific repeat code, the RAN proteins consist of iterations of one, two, four, or five amino acids (Table 1). RAN proteins consisting of two amino acid repeats are called “dipeptide repeat proteins” (DPRs; Mackenzie *et* *al,* 2013). The presence of the repeat expansion might also lead to loss of function of the respective protein. This can be caused by decreased transcription initiation (e.g., epigenetic alterations), defective transcription (e.g., abortion), or increased mRNA degradation of the host gene (Todd *et* *al,* 2010; Haeusler *et* *al,* 2014). In the next chapter, we will give an overview of the current state of evidence regarding which mechanism is at play (RNA toxicity, RAN toxicity, and loss of function) in C9 ALS/FTD and the other non‐coding repeat expansion disorders.

## Mechanisms in C9 ALS/FTD

Amyotrophic lateral sclerosis (ALS) is an adult‐onset neurodegenerative disease characterized by progressive degeneration of upper and lower motor neurons (Swinnen & Robberecht, 2014). Clinically, patients present with painless subacute focal muscle weakness. The disease is rapidly progressive, generally leading to death in 3–5 years after symptom onset, and is unfortunately still incurable (Swinnen & Robberecht, 2014). The genetic landscape of ALS has been redrawn significantly in recent years. In most (± 90%) cases, ALS does not run in the family and is hence called “sporadic ALS” (sALS). The remainder (± 10%) of ALS patients, however, has an affected first degree, which is called “familial ALS” (fALS). In the latter, a monogenetic cause is evidently suspected. Indeed, such monogenetic mutation is identified in the majority (± 70%) of patients, with *C9ORF72*,* FUS*,* TARDBP,* and *SOD1* being the most frequent ones (Renton *et* *al,* 2014). Surprisingly, a monogenetic cause is identified in ± 10% of sALS patients, most likely reflecting both *de novo* mutations and incomplete penetrance of mutations. Frontotemporal dementia (FTD) is the clinical dementia syndrome caused by frontotemporal lobe degeneration (FTLD) and is the second most common dementia after Alzheimer's disease (AD) in patients younger than 65 years (Olney *et* *al,* 2017).

**Figure 2:**
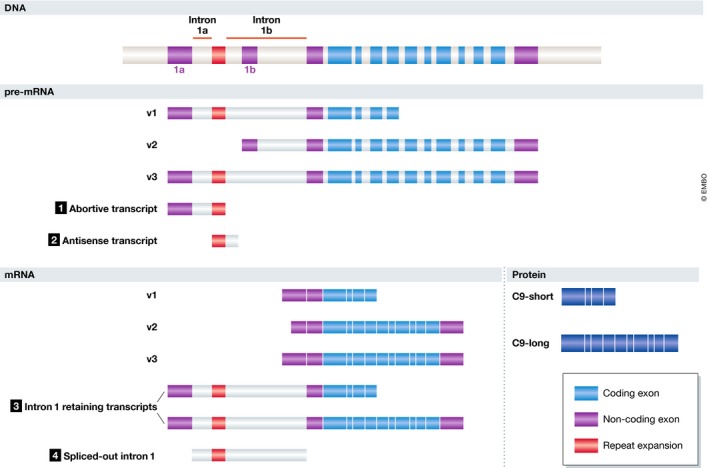
C9ORF72 gene structure, transcription, and translation Four potentially pathogenic RNA species can be discerned. (1) At the pre‐mRNA level, transcription of v1 and v3 might stall at the repeat region, resulting in the generation of abortive transcripts. (2) Transcription of the repeat region in the antisense direction generates antisense transcripts. (3) Ineffective splicing of intron 1 in transcripts v1 and v3 might result in intron 1‐retaining transcripts. (4) Effective splicing of intron 1 in transcripts v1 and v3 might generate repeat‐containing spliced‐out intron 1.

Amyotrophic lateral sclerosis and FTD are considered to constitute the extremes of a disease spectrum (Swinnen & Robberecht, 2014). The most frequent cause of ALS/FTD is a repeat expansion in the *C9ORF72* gene (DeJesus‐Hernandez *et* *al,* 2011; Renton *et* *al,* 2011, 2014). Its structure at the DNA, RNA, and protein level is depicted in Fig 2. *Post‐mortem* examinations of C9 ALS/FTD cases reveal TDP‐43 pathology (Mackenzie *et* *al,* 2014; Saberi *et* *al,* 2015), reminiscent of sporadic ALS cases, but also RAN proteins (i.e., dipeptide repeat proteins (DPRs)) (Zu *et* *al,* 2013) and RNA foci (DeJesus‐Hernandez *et* *al,* 2011).

### C9 ALS/FTD is mainly a gain‐of‐function disease

C9 ALS/FTD is considered to be mainly driven by a gain‐of‐function mechanism, based on several observations. First, patients homozygous for the repeat expansion do not have an excessively aggressive clinical nor pathological phenotype which would have been expected in case of a loss‐of‐function mechanism (Cooper‐Knock *et* *al,* 2013; Fratta *et* *al,* 2013). Second, while there is one study reporting a *C9ORF72* coding mutation (Liu *et* *al,* 2016), none were found in a large cohort of ALS patients (Harms *et* *al,* 2013). Third, *C9ORF72* promoter hypermethylation, associated with gene silencing, is neuroprotective as observed using cross‐sectional and longitudinal neuroimaging data (McMillan *et* *al,* 2015). Fourth, several *in vitro* observations are not in line with a loss‐of‐function hypothesis. Most importantly, *C9ORF72* transcript‐directed antisense oligonucleotide (ASO) treatment resulting in decreased or dysfunctional *C9ORF72* transcripts rescued the phenotype (e.g., glutamate‐induced cell death (Donnelly *et* *al,* 2013) and transcriptional changes (Sareen *et* *al,* 2013), cfr. Table 5) in patient‐derived induced motor neurons (iMNs). Furthermore, ASO‐mediated *C9ORF72* knockdown has no effect in control iMNs and neuronal primary cultures (Sareen *et* *al,* 2013; Sellier *et* *al,* 2016). Fifth, none of the *C9orf72* knockout murine models develop a neurodegenerative phenotype (Lagier‐Tourenne *et* *al,* 2013; Koppers *et* *al,* 2015; Atanasio *et* *al,* 2016; Burberry *et* *al,* 2016; Jiang *et* *al,* 2016; O'Rourke *et* *al,* 2016; Sudria‐Lopez *et* *al,* 2016). Altogether, these data support the conclusion that *C9ORF72* loss‐of‐function is not the main pathogenic driver suggesting mainly a gain‐of‐function mechanism; i.e., RNA and/or RAN toxicity.

### RNA toxicity in C9 ALS/FTD

The exact nature of the repeat RNA present in RNA foci is still unclear. Four RNA species can be proposed (Fig 2). At the pre‐mRNA level, transcription of transcripts v1 and v3 might stall at the repeat region, resulting in the generation of abortive transcripts. Transcription of the repeat region in the antisense direction also generates antisense transcripts. Ineffective splicing of intron 1 in transcripts v1 and v3 might result in intron 1‐retaining transcripts. Finally, effective splicing of intron 1 in transcripts v1 and v3 might generate repeat‐containing spliced‐out intron 1. In general, repeat RNA is thought to form RNA foci that contain a cluster of repeat RNAs in complex with several RNA‐binding proteins (Kumar *et* *al,* 2017). RNA foci are not restricted to neurons and are also found in astrocytes, microglia, and oligodendrocytes (Mizielinska *et* *al,* 2013). While RNA foci are mostly intranuclear, cytoplasmic RNA foci as well as RNA foci at the edge of the nucleus have also been observed (Mizielinska *et* *al,* 2013; Cooper‐Knock *et* *al,* 2015b). RNA foci do not follow a rostrocaudal anatomical distribution as they are equally prevalent in the frontal cortex and in the spinal cord (DeJesus‐Hernandez *et* *al,* 2011, 2017).

### RAN (DPR) toxicity in C9 ALS/FTD

Both sense DPRs (GA, GR, GP) and antisense DPRs (PR, PA, GP) are formed with sense DPRs being more abundant than antisense and GA being the most frequently observed one (Mori *et* *al,* 2013a; Mackenzie *et* *al,* 2015). DPRs are exclusively present in neurons and are mainly detected as cytoplasmic aggregates (Ash *et* *al,* 2013; Mackenzie *et* *al,* 2013). All DPRs have a similar anatomical distribution and are most abundant in cortical and cerebellar regions and almost absent in brainstem and spinal cord (Davidson *et* *al,* 2016; Mackenzie *et* *al,* 2015).

The toxic potential of the different DPRs has been examined comprehensively both in *in vitro* and *in vivo* disease models (Table 2). The potential mechanisms of this DPR toxicity have recently been reviewed (Freibaum & Taylor, 2017). Altogether, these data indicate that the arginine‐rich DPRs can be highly toxic, at least in overexpression systems. Data also support the notion that GA can be toxic, while GP and PA are probably harmless (at least in the currently available disease models). Despite these *in vitro* and *in vivo* findings, it remains to be determined whether DPRs contribute to the pathogenesis of C9 ALS/FTD in humans. One should note that obtaining *post‐mortem* support for DPR toxicity might be difficult as toxic DPR species might kill vulnerable motor neurons, hence leaving no trace to be uncovered. However, recent *post‐mortem* data favor an association between DPRs and pathology as GR aggregates correlate with neurodegeneration and even colocalize with phospho‐TDP‐43, albeit with some variability (Saberi *et* *al,* 2018; Sakae *et* *al,* 2018). Moreover, whereas DPR load generally does not correlate with clinical severity (Gendron *et* *al,* 2015), cerebellar GP levels inversely correlate with cognitive scores and GA burden is inversely related with age at onset (Davidson *et* *al,* 2014). Nevertheless, several *post‐mortem* observations are difficult to reconcile with DPR toxicity being the main culprit. Anatomical distribution of DPR aggregation *post‐mortem* does not obviously correlate with neurodegeneration. In short, DPR load is highest in unaffected tissue (i.e., cerebellum) and lowest in affected tissue (i.e., spinal motor neurons; Gomez‐Deza *et* *al,* 2015; Mackenzie *et* *al,* 2015). Moreover, coexistence of DPR and TDP‐43 aggregates in a given cell is very rare (Mackenzie *et* *al,* 2013; Davidson *et* *al,* 2014). In addition, their predominant appearance in disease models is not in line with *post‐mortem* findings (Mackenzie *et* *al,* 2015), especially the supposed nucleolar localization of GR and PR in disease models (Kwon *et* *al,* 2014; Wen *et* *al,* 2014). Nevertheless, the *in vitro* and *in vivo* models where GA forms cytoplasmic aggregates recapitulate *post‐mortem* findings in C9 ALS/FTD patients (Mackenzie *et* *al,* 2015). Altogether and despite the DPR toxicity observed in both *in vitro* and *in vivo* systems, its pathogenic involvement in ALS is still an open question.

**Table 2:**
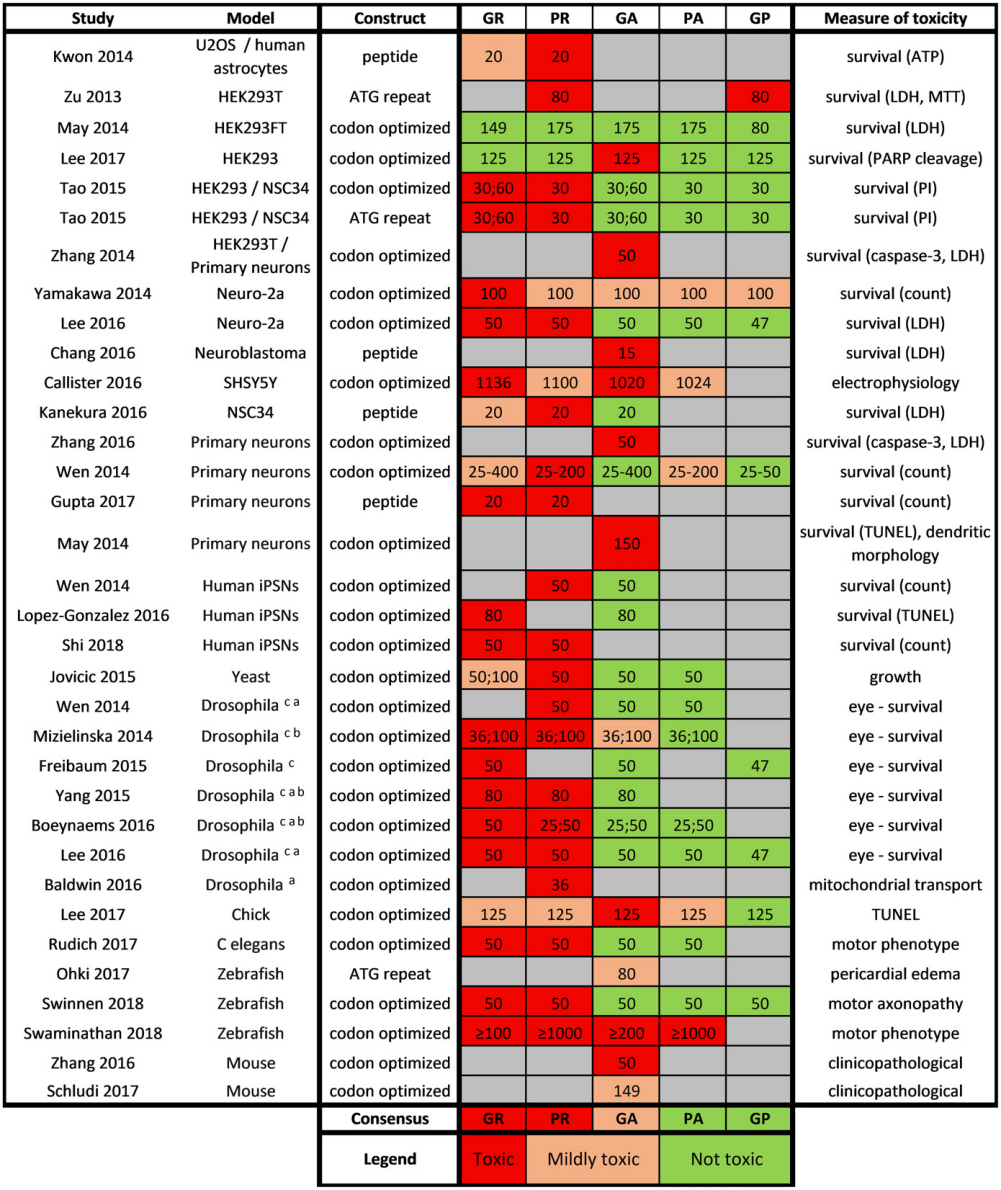
*In vitro* and *in vivo* toxicity of individual DPRs

Numbers indicate the repeat lengths used.

*Abbreviations*: ATP, adenosine triphosphate; LDH, lactate dehydrogenase; MTT, 3‐(4,5‐dimethylthiazol‐2‐yl)‐2,5‐diphenyltetrazolium bromide; PI, propidium iodide; TUNEL, terminal deoxynucleotidyl transferase dUTP nick end labeling.

Motor neuron driver (OK371 or D42).

Pan‐neuronal driver (elav or tubulin).

Eye driver (GMR).

### Loss of function in C9 ALS/FTD

Despite C9 ALS/FTD being considered as mainly having a gain‐of‐function disease mechanism, some data indicate that *C9ORF72* loss‐of‐function might contribute to disease pathogenesis and could enhance the gain‐of‐function mechanisms. In C9 ALS/FTD *post‐mortem* brain tissue, *C9ORF72* transcript levels are decreased by 50% (DeJesus‐Hernandez *et* *al,* 2011; Gijselinck *et* *al,* 2012; van Blitterswijk *et* *al,* 2015). Similarly, decreased levels of the long C9orf72 protein isoform have been observed in the frontal and temporal cortex of C9 ALS/FTD patients (Waite *et* *al,* 2014; Xiao *et* *al,* 2015; Saberi *et* *al,* 2018). Knockdown of *C9ORF72* in *in vitro* models is associated with autophagic dysfunction, including p62 accumulation, perinuclear clustering of swollen lysosomes, and TDP‐43 aggregation (Sellier *et* *al,* 2016; Webster *et* *al,* 2016; Yang *et* *al,* 2016; Amick & Ferguson, 2017; Aoki *et* *al,* 2017). In patient‐derived cells, the glutamate hypersensitivity phenotype is rescued by *C9ORF72* overexpression as well as being recapitulated by *C9ORF72* knockout in control cells (Shi *et* *al,* 2018). The mechanism of *C9ORF72* loss of function as well as its contribution to disease pathogenesis has recently been reviewed in detail (Balendra & Isaacs, 2018). Essentially, *C9ORF72* loss‐of‐function might contribute to pathology via its role in autophagy (Balendra & Isaacs, 2018; Webster *et* *al,* 2018).

## Mechanisms in other repeat expansion diseases

### Myotonic dystrophy type 1

Myotonic dystrophy type 1 is caused by CTG repeats in the 3′ UTR of *DMPK* and is mainly driven by RNA toxicity. CUG repeat RNA adopts a stable hairpin conformation (Tian *et* *al,* 2000) that forms nuclear RNA foci (Taneja *et* *al,* 1995; Davis *et* *al,* 1997). The RNA foci can sequester muscleblind‐like (MBNL) proteins (Miller *et* *al,* 2000; Mankodi *et* *al,* 2001) leading to an imbalance between MBNL proteins and CUGBP1 (Lin *et* *al,* 2006; Kuyumcu‐Martinez *et* *al,* 2007). This imbalance causes altered splicing of several mRNAs (e.g., the insulin receptor *IR2*, the chloride channel *CLC2,* and the cardiac troponin *cTNNT2*) in a tissue‐dependent manner (Philips *et* *al,* 1998; Charlet‐B *et* *al,* 2002; Mankodi *et* *al,* 2002; Fugier *et* *al,* 2011) explaining the various multisystemic phenotypic features. Missplicing has been confirmed in patient tissue and correlates with clinical features (Savkur *et* *al,* 2001; Fugier *et* *al,* 2011; Freyermuth *et* *al,* 2016), and MBNL1 dysfunction is regarded as the key mechanism involved in myotonic dystrophy 1. Observations in several mouse models [i.e., *Mbnl1* knockout (Kanadia *et* *al,* 2003), *Mbnl2* knockout (Hao *et* *al,* 2008), *Cugbp1* overexpressing (Ho *et* *al,* 2005; Ward *et* *al,* 2010), and (CUG)n expressing (Mahadevan *et* *al,* 2006)] are consistent with this view. However, RNA toxicity might encompass more than missplicing alone. Several additional modes of action of CUG repeat RNA toxicity have been proposed, including miRNA misprocessing (Perbellini *et* *al,* 2011), transcriptional dysregulation (Botta *et* *al,* 2007), global translational inhibition through stress granule induction (Onishi *et* *al,* 2008; Huichalaf *et* *al,* 2010), and use of alternative polyadenylation sites (Batra *et* *al,* 2014). In addition to RNA toxicity, RAN toxicity has been suggested as well. While polyQ, derived from antisense CAG repeat RNA, has been found in patient material (Zu *et* *al,* 2011), its pathogenic contribution is still not clear. *DMPK* loss of function is unlikely given the absence of a clear relevant phenotype in *Dmpk* knockout mice (Jansen *et* *al,* 1996; Reddy *et* *al,* 1996).

### Myotonic dystrophy type 2

Myotonic dystrophy type 2 is caused by CCTG repeats in the intron of *ZNF9* and resembles myotonic dystrophy type 1 in many regards. As a consequence, the underlying mechanism is believed to be very similar as well. Essentially, the CCUG repeat RNA leads to an MBNL‐CUGBP1 imbalance (Salisbury *et* *al,* 2009; Jones *et* *al,* 2011), making RNA toxicity the prevailing mechanism. However, as both sense (LPAC) and antisense (QAGR) RAN peptides are present in *post‐mortem* tissue and display *in vitro* toxicity (Zu *et* *al,* 2017), they might contribute to certain aspects of the disease as well. Additionally, CNBP loss of function might also play a role, as *Cnbp*‐deficient mice develop key features of myotonic dystrophy (Chen *et* *al,* 2007).

### Fragile X tremor ataxia syndrome

FXTAS is caused by CGG repeats in the 5′ UTR of the *FMR1* gene. Pathological hallmarks of FXTAS consist of Purkinje cell loss and intranuclear ubiquitin‐positive inclusions containing a polyglycine RAN peptide (Buijsen *et* *al,* 2014; Boivin *et* *al,* 2018). Loss of function is excluded as patients with Fragile X syndrome, caused by *FMR1* loss‐of‐function due to very long (> 200) CGG repeats, do not develop any FXTAS features (Boivin *et* *al,* 2018). Moreover, *FMR1* mRNA levels are even increased in FXTAS patients (Kenneson *et* *al,* 2001; Allen *et* *al,* 2004) and expression of CGG repeat RNA induces *in vitro* and *in vivo* neurotoxicity (Jin *et* *al,* 2003; Willemsen *et* *al,* 2003; Hukema *et* *al,* 2014), suggesting a primary gain‐of‐function mechanism. The CGG repeat RNA is able to adopt secondary structures (i.e., G‐quadruplexes, duplexes and hairpins; Malgowska *et* *al,* 2014), which might compromise the function of various RNA‐binding proteins like Pur‐alpha (Jin *et* *al,* 2007), hnRNPA2/B1 (Sofola *et* *al,* 2007), CUGBP1 (Sofola *et* *al,* 2007), Sam68 (Sellier *et* *al,* 2010), and Drosha‐DGCR8 (Sellier *et* *al,* 2013). The observation that overexpression of most of these proteins can rescue the phenotype in CGG *Drosophila* (Jin *et* *al,* 2007; Sofola *et* *al,* 2007; Sellier *et* *al,* 2013) supports a functional role of these proteins in FXTAS pathogenesis. However, RNA toxicity seems to be insufficient to explain FXTAS pathogenesis because of the following three observations. First, the repeat size is relatively short, compared to (mainly) RNA toxicity driven diseases (e.g., myotonic dystrophy—cfr. Table 1). Second, the large ubiquitin‐positive intranuclear inclusions in FXTAS are reminiscent of aggregates typically seen in protein‐mediated neurodegenerative disorders (e.g., Huntington's disease). Third, the toxicity of CGG constructs in *Drosophila* and mouse models seems to depend on FMRpolyG production (Todd *et* *al,* 2013; Sellier *et* *al,* 2017) suggesting a contribution of RAN toxicity. FMRpolyG has been found in patient‐derived cells (Sellier *et* *al,* 2017), mouse models (Hukema *et* *al,* 2015; Sellier *et* *al,* 2017) and in *post‐mortem* tissue (Todd *et* *al,* 2013; Buijsen *et* *al,* 2014), where it colocalizes with the ubiquitin‐positive intranuclear inclusions (Todd *et* *al,* 2013). In several models, FMRpolyG displays a length‐dependent propensity to aggregate in the nucleus (Todd *et* *al,* 2013; Sellier *et* *al,* 2017), and it is suggested to be neurotoxic by disturbing the ubiquitin‐proteasome system (UPS) and the nuclear lamina structure (Oh *et* *al,* 2015; Sellier *et* *al,* 2017). Interestingly, antisense RAN proteins have also been observed in patient material (Krans *et* *al,* 2016). However, their pathogenic contribution has not yet been characterized.

### SCA8

The 3′UTR repeat expansion in SCA8 is bidirectionally transcribed (i.e., (CTG.CAG)n), complicating the quest for the underlying mechanism. In the *ATXN8* strand, the CUG repeat RNA is believed to cause RNA toxicity via MBNL1 dysfunction, similar to what is seen in myotonic dystrophy. Supporting this, nuclear CUG RNA foci colocalize with MBNL1 in molecular layer interneurons of SCA8 patients and mouse models, and loss of *Mbnl1* exacerbates the phenotype of SCA8 mice (Daughters *et* *al,* 2009). Additionally, splicing changes in a target of MBNL1 (i.e., GAT4) have been established in *post‐mortem* tissue, validating the pathogenic relevance of RNA toxicity (Daughters *et* *al,* 2009). In the *ATXN8OS* strand, the CAG repeat RNA is translated into toxic polyglutamine and intranuclear polyglutamine inclusions have been seen in Purkinje cells and brainstem neurons of SCA8 mice and *post‐mortem* tissue (Moseley *et* *al,* 2006). Moreover, in *post‐mortem* tissue polyserine derived from the *ATXN8OS* strand by RAN translation has been discovered in degenerating white matter regions (Ayhan *et* *al,* 2018). Loss of function of the host gene(s) seems unlikely, as individuals harboring a genomic deletion in the SCA8 region do not exhibit cerebellar degeneration (Mandrile *et* *al,* 2016). Moreover, CTG expression in *Drosophila* and mouse models leads to neurodegenerative phenotypes further supporting a gain‐of‐function mechanism (Moseley *et* *al,* 2006; Tripathi *et* *al,* 2016). Therefore, SCA8 seems to be mainly driven by two gain‐of‐function mechanisms, being RNA and RAN toxicity, arising from bidirectional repeat RNAs.

### SCA10

SCA10 is caused by ATTCT repeats in the intron of *ATXN10,* and a gain‐of‐function mechanism has been proposed due to two main observations. First, an *ATXN10* loss‐of‐function mechanism is unlikely as *ATXN10* transcript levels are unaltered in SCA10 patients (Wakamiya *et* *al,* 2006), as heterozygous *Atxn10* knockout mice display no abnormalities (Wakamiya *et* *al,* 2006) and as loss‐of‐function *ATXN10* mutations do not give rise to a SCA10 phenotype in humans (Keren *et* *al,* 2010). Second, *in vitro* and *in vivo* (mainly mouse) models overexpressing ATTCT repeat constructs exhibit phenotypes resembling SCA10 (White *et al*, 2010; White *et* *al,* 2012). The exact nature of this gain of function is still unclear, but current data suggest RNA toxicity as the prevailing mechanism. The repeat expansion is spliced out and adopts a hairpin structure (Handa *et* *al,* 2005; Park *et* *al,* 2015) that binds hnRNPK *in vitro* and forms nuclear and cytoplasmic RNA foci in patient‐derived cells which colocalize with hnRNPK (White *et* *al,* 2010). Furthermore, hnRNPK overexpression rescues *in vitro* ATTCT toxicity (White *et* *al,* 2010), indicating that hnRNPK dysfunction is a key factor in SCA10. However, *post‐mortem* examinations have not been performed yet and RAN peptides (i.e., poly(ILFYS)) have not been assessed leaving the role of RAN toxicity in SCA10 unclear.

### SCA12

SCA12 is caused by CAG repeats in the promoter region of *PPP2R2B* that encodes a subunit of the phosphatase PP2A. The repeat is located in the promoter region of one (of many) protein isoforms, leading to increased promoter activity upon repeat expansion (O'Hearn *et* *al,* 2015). Overexpression of *PPP2R2B* is toxic both *in vitro* (O'Hearn *et* *al,* 2015) and in *Drosophila* (Wang *et* *al,* 2011) suggesting gain‐of‐function toxicity. However, *PPP2R2B* mRNA and protein levels have not yet been assessed in *post‐mortem* tissue making the *PPP2R2B* gain‐of‐function mechanism still hypothetical. The contribution of RNA and RAN toxicity has also not been evaluated in *post‐mortem* tissue, and suitable disease models are also lacking. Nevertheless, SCA12 is unlikely to be a polyglutamine disease, as polyglutamine inclusions are absent in a *post‐mortem* case (O'Hearn *et* *al,* 2015). The disease phenotypes are also rather mild compared to other polyglutamine diseases.

### SCA31

SCA31 is caused by intronic TGGAA repeats in the *BEAN1* and *TK2* genes. Small nuclear sense RNA foci are present exclusively in Purkinje cells (Niimi *et* *al,* 2013) and colocalize with TDP‐43 (Ishiguro *et* *al,* 2017). Repeat RNA‐binding proteins include TDP‐43, SRSF1, SRSF9, NONO, Matrin3, and several hnRNPs (Sato *et* *al,* 2009; Ishiguro *et* *al,* 2017). *In vitro* and *in vivo* (*Drosophila*) expression of TGGAA repeat constructs leads to toxicity that is suppressed by overexpression of the RNA‐binding proteins TDP‐43, hnRNPA2, and FUS, supporting an RNA toxicity gain‐of‐function mechanism (Niimi *et* *al,* 2013; Ishiguro *et* *al,* 2017). Nevertheless, poly(WNGME) has been detected as granular cytoplasmic inclusions in Purkinje cells and in *Drosophila* (Ishiguro *et* *al,* 2017). Moreover, production of poly(WNGME) in the latter was reduced upon TDP‐43 overexpression (Ishiguro *et* *al,* 2017). Therefore, RAN toxicity in SCA31 cannot be excluded at this moment.

### SCA36

SCA36 is caused by intronic TGGGCC expansions in *NOP56,* and sense RNA foci are abundantly present throughout the brain (Liu *et* *al,* 2014). Interestingly, antisense RNA foci have not been observed in *post‐mortem* tissue nor in a mixed neuronal population derived from induced pluripotent stem cells (iPSCs; Matsuzono *et* *al,* 2017). SRSF2, an RNA‐binding protein that mainly functions as a splicing factor, binds to UGGGCC repeat RNA and colocalizes with RNA foci in patient‐derived lymphoblasts (Kobayashi *et* *al,* 2011). The role of RAN toxicity in SCA36 is difficult to evaluate based on present data. While a first report did not observe any neuronal inclusions of ubiquitin or p62 (Obayashi *et* *al,* 2015), these were present in a second case (Liu *et* *al,* 2014), predominantly in the inferior olivary nucleus.

### Huntington's disease‐like 2

Huntington's disease‐like 2 is caused by CTG repeats in an alternatively spliced exon of *JPH3*. The disease clinically and pathologically mimics Huntington's disease. In *post‐mortem* tissue, corticostriatal degeneration and intranuclear ubiquitin‐positive polyglutamine inclusions are evident (Greenstein *et* *al,* 2007; Rudnicki *et* *al,* 2008). Given what we know about Huntington's disease, RAN toxicity is likely to be the prevailing mechanism in HDL‐2, driven by polyglutamine generated from antisense CAG repeat RNA. This is supported by the observation of antisense transcripts as well as polyglutamine inclusions in an HDL‐2 mouse model (Wilburn *et* *al,* 2011). Nevertheless, protein toxicity mediated by polyalanine or polyleucine in the sense direction through canonical translation cannot be excluded. RNA toxicity driven by CUG repeat RNA, supposedly through MBNL1 dysfunction, might also be at play. As RNA foci are present in *post‐mortem* tissue and as HDL‐2 repeat RNA is toxic *in vitro* (Rudnicki *et* *al,* 2007), more work still needs to be done to investigate the role of RNA toxicity in HDL‐2. Loss of function of the *JPH3* gene might also contribute as JPH3 protein levels are decreased in *post‐mortem* samples and as *jph3* knockout mice develop a motor phenotype (Seixas *et* *al,* 2012).

## Disentangling RNA and RAN toxicity: a Gordian knot in C9 ALS/FTD?

**Figure 3:**
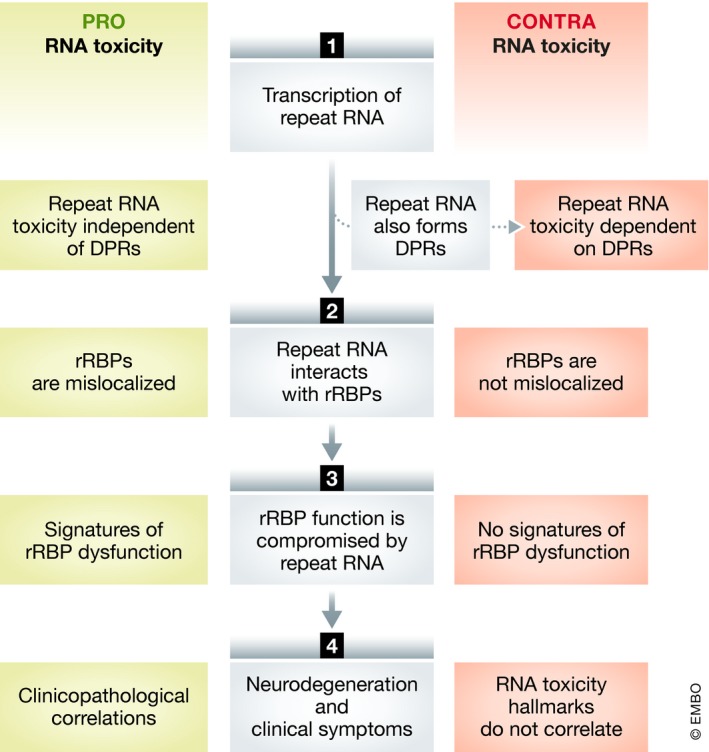
Roadmap to prove/disprove RNA toxicity Arguments pro/contra RNA toxicity can be generated at four levels of the presumed pathogenic cascade of RNA toxicity.

In order to further understand the contribution of RNA and RAN toxicity, we will focus on the C9 ALS/FTD paradigm. Recent research into C9 ALS/FTD has generated many disease models that allow for a more complete evaluation of the contribution of both mechanisms to pathology. Unfortunately, disentangling these two mechanisms in disease models is very difficult (cfr. Box 1). To specifically assess the contribution of RNA toxicity in the pathogenesis of C9 ALS/FTD, several approaches can be employed, each targeting a different step in the pathway leading to RNA toxicity (Fig 3). We will systematically discuss the current state of evidence at these different levels.

**Figure 4:**
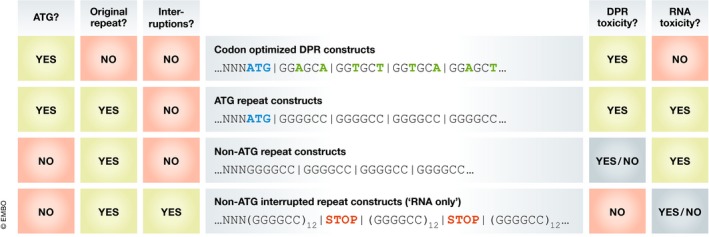
Constructs used to model C9ORF72 gain‐of‐function toxicity First, codon‐optimized DPR constructs generate DPRs but do not have the potential to inflict RNA toxicity since the lack of a repetitive sequence. Therefore, these constructs allow an easy modeling of DPR toxicity. Second, ATG repeat constructs can theoretically induce RNA toxicity but by default also generate DPRs. Therefore, RNA toxicity cannot be investigated with these constructs. Third, repeat constructs lacking an ATG start codon (i.e., “non‐ATG repeat constructs”) can also give rise to RNA toxicity while DPR generation is uncertain since it needs to rely on RAN translation. Therefore, by assessing the presence of DPRs these constructs can be used to assess RNA toxicity. Fourth, so‐called “RNA only” constructs should theoretically only give rise to RNA toxicity as the repeat sequence is regularly interrupted by stop codons interfering with RAN translation.

**Box 1. The enigma of modeling RNA toxicity with “non‐ATG repeat constructs” (with Fig 4)**

Proving that repeat RNA exerts its toxicity independent of DPR formation is very challenging due to intrinsic methodological limitations of the constructs used to model *C9ORF72* gain of function (Fig 4). Theoretically, four different types of constructs can be used, each potentially modeling DPR and/or RNA toxicity (Fig 4). The origin of potential toxicity is clear for “codon‐optimized” and “RNA only” constructs (i.e., DPRs and RNA, respectively). “ATG repeat constructs” cannot be used to model RNA toxicity as DPRs are generated by default, hence obscuring any potential RNA toxicity. For “non‐ATG repeat constructs,” the situation is difficult as DPRs can only be generated by RAN translation and hence are not present by default. Therefore, if no DPRs are detected, toxicity is to be attributed to the repeat RNA itself, indicating RNA toxicity. As such, a good DPR detection approach is important. Three major aspects are important to consider (for a given study, this is covered under the headings ‘Adequate methodology?’ in Tables 3, 4, 5). First, the DPR detection method needs to have a high sensitivity and specificity. Only highly sensitive DPR detection methods (i.e., ELISA or dot blot, opposed to immunohistochemistry or Western blot) have sufficient power to confidently assess DPR presence. Specificity relies on the use of appropriate positive and negative controls. Second, the presence of all possible DPRs should be investigated. Third, (raw) data concerning DPR detection should be provided. Additionally, these models should display C9 ALS/FTD hallmarks in order to claim disease relevance (i.e., RNA foci, TDP‐43 pathology, or motor neuron degeneration).

**Table 3:**
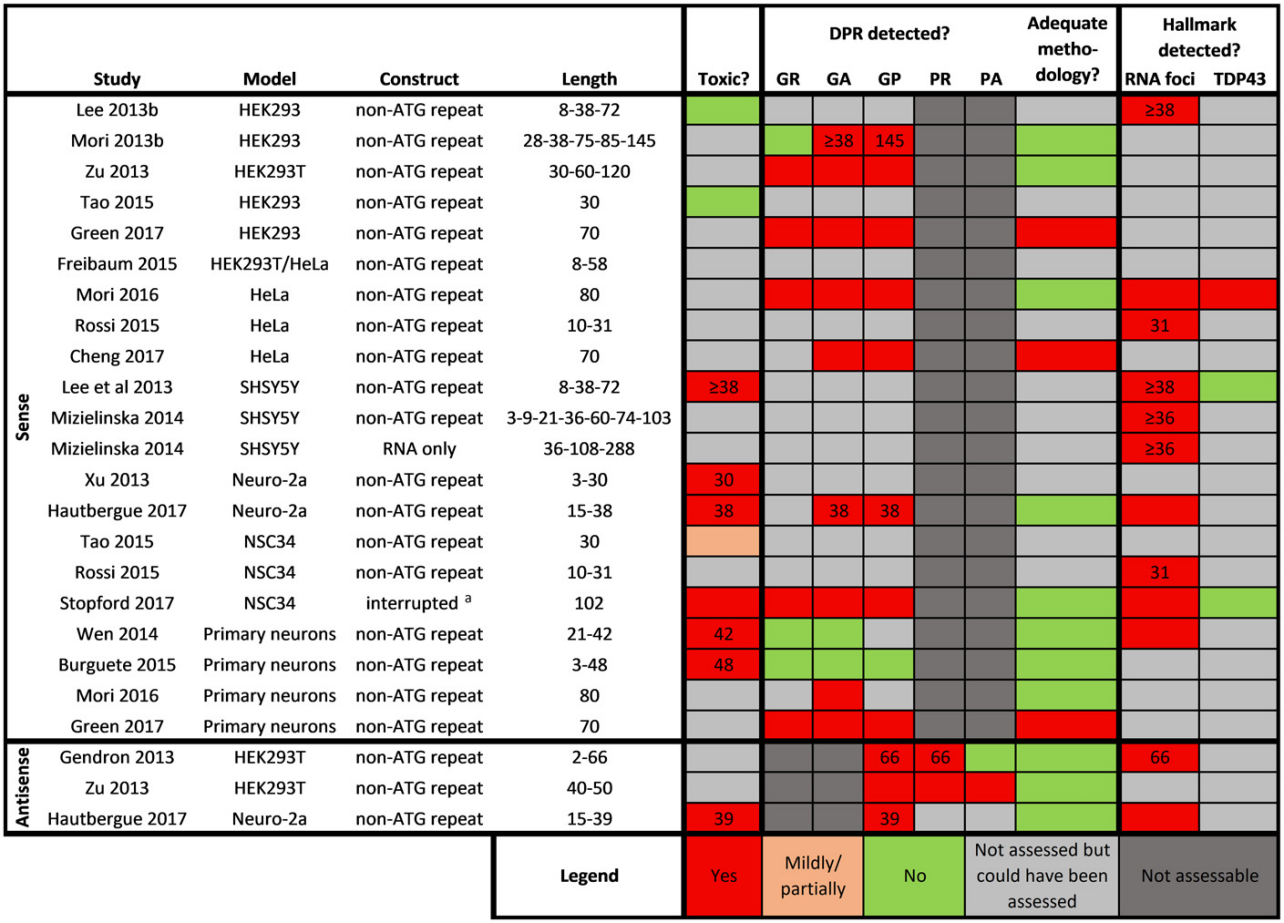
Mammalian cell culture non‐ATG C9ORF72 repeat models

Overview of toxicity, detection of DPRs, and presence of C9ORF72 ALS hallmarks in sense and antisense *in vitro* models expressing non‐ATG C9ORF72 repeat constructs. If a modality was only found to be present at certain repeat lengths, these are indicated as numbers in the respective boxes.

Non‐stop‐codon interrupted.

**Table 4:**
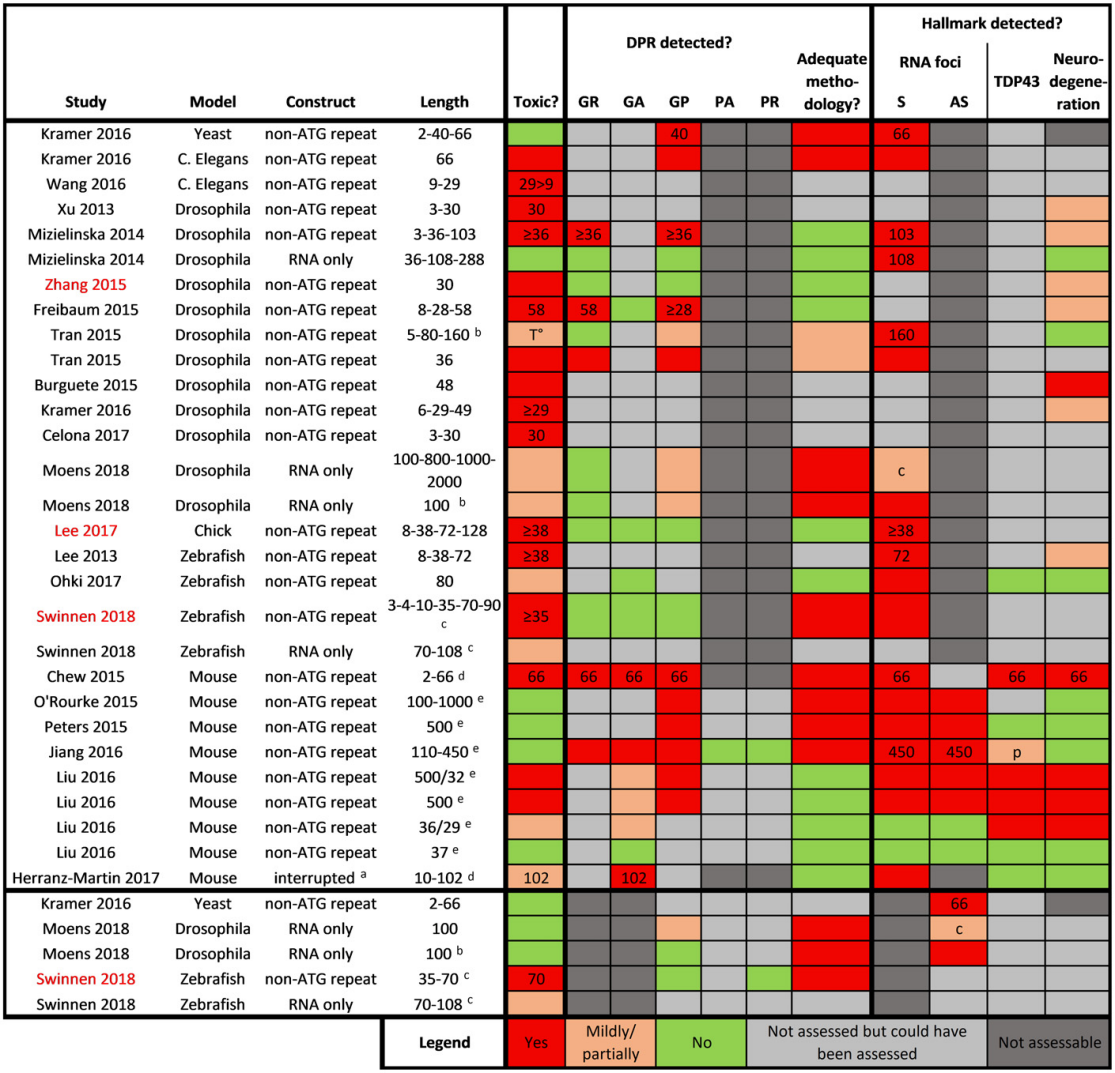
*In vivo* non‐ATG C9ORF72 repeat models

Overview of toxicity, detection of DPRs, and presence of C9 ALS hallmarks in sense and antisense *in vivo* models expressing non‐ATG C9ORF72 repeat constructs. Studies in red indicate those that found toxicity in the absence of DPRs. If a modality was only found to be present at certain repeat lengths, these are indicated as numbers in the respective boxes.

*Abbreviations*: c, cytoplasmic; p, increased levels of phospho‐TDP43; T°, only toxic upon higher temperature.

Non‐stop‐codon interrupted.

Intronic.

RNA microinjection.

AAV intracerebroventricular injection.

BAC.

**Table 5:**
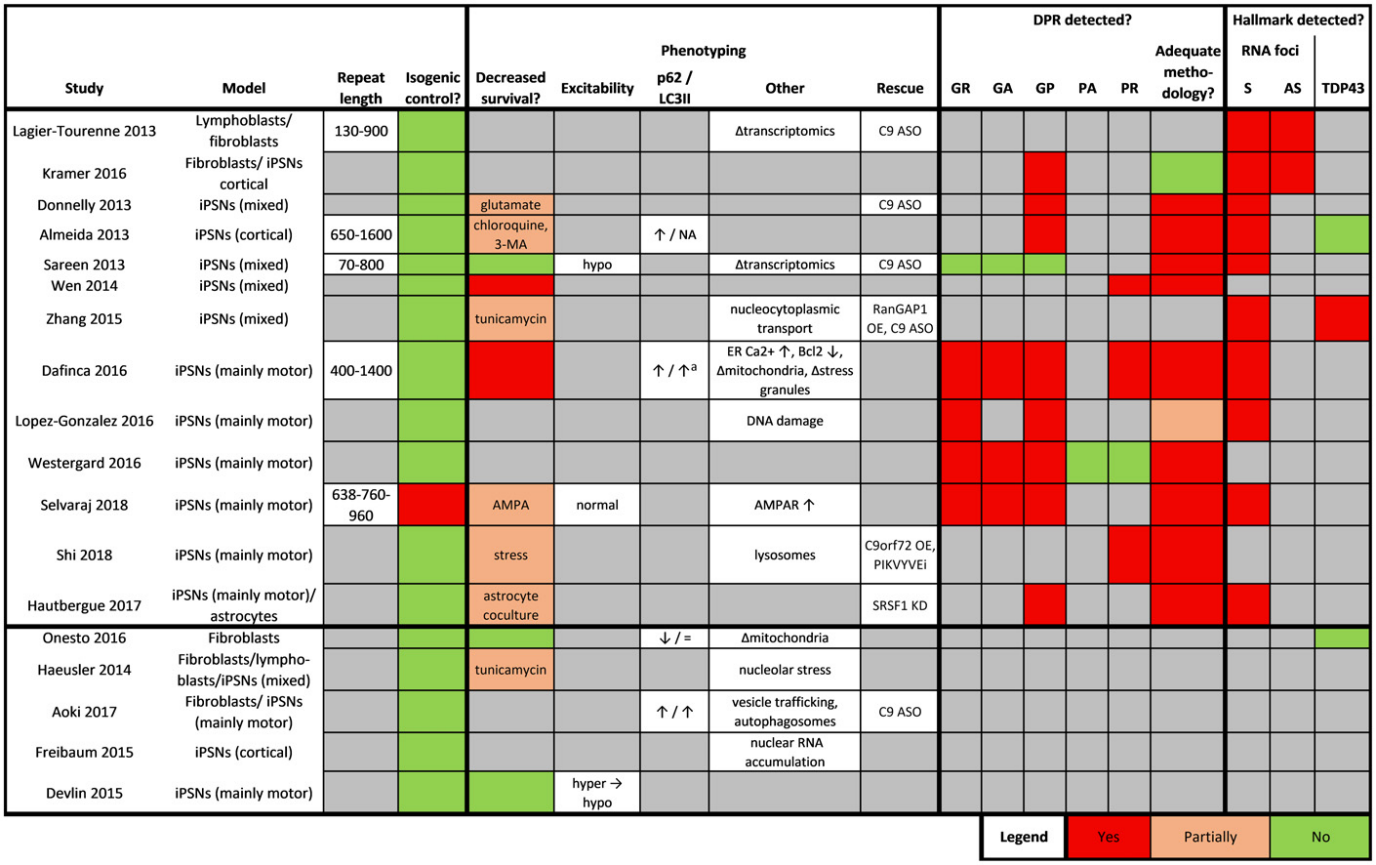
Patient‐derived cellular *in vitro* C9ORF72 models

Systematic overview of all studies employing and characterizing patient‐derived *in vitro* models for C9ORF72 ALS/FTD. For each model, repeat length, use of isogenic control, phenotyping, DPR detection, RNA foci, and TDP43 pathology are reported systematically. Upper part of table contains studies assessing DPR and/or RNA foci presence. Four studies (Burguete *et* *al,* 2015; Mori *et* *al,* 2016; Niblock *et* *al,* 2016; Webster *et* *al,* 2016) were excluded because no characterization was performed. If a survival phenotype was observed upon additional treatment, this treatment is indicated.

*Abbreviations*: AS, antisense; ASO, antisense oligonucleotide; ER, endoplasmic reticulum; iPSNs, induced pluripotent stem cell‐derived neurons; KD, knockdown; NA, not assessed; OE, overexpression; S, sense.

Increased in cortical neurons, not in motor neurons.

### RNA toxicity in the absence of DPRs in C9ORF72 hexanucleotide disease models

#### Mammalian cell culture non‐ATG repeat models

Only a minority of *in vitro* studies have performed a full and complete characterization of hexanucleotide repeat expansion (HRE) models (Table 3). A key problem concerns the DPR detection methodology in many of the studies. Four studies assessed the relationship between toxicity and DPR formation (Wen *et* *al,* 2014; Burguete *et* *al,* 2015; Hautbergue *et* *al,* 2017; Stopford *et* *al,* 2017). Two of these studies found repeat toxicity to occur in the absence of detecting DPRs, suggesting an involvement of RNA toxicity (Wen *et* *al,* 2014; Burguete *et* *al,* 2015). However, the DPR detection methodology might not be optimal in order to draw strong conclusions (low sensitivity with inadequate/absent positive control).

#### *In vivo* non‐ATG repeat models

Different *in vivo* HRE models have been characterized also with respect to the presence of DPRs (Table 4). Altogether, three studies found toxicity in the absence of DPR detection, suggestive of RNA toxicity. First, a *Drosophila* model expressing (GGGGCC)_30_ in the eye or in adult neurons showed toxicity without detection of GR and GP (Zhang *et* *al,* 2015). Unfortunately, a non‐ideal positive control was used (i.e., construct under control of a different promoter and different treatment condition) and the presence of GA was not assessed. In a second study, GGGGCC repeats induced toxicity in a chicken embryo model without obvious presence of DPRs as assessed by immunohistochemistry (Lee *et* *al,* 2017). Third, using a zebrafish model we observed GGGGCC repeat RNA to induce motor axonal toxicity in the absence of DPRs (Swinnen *et* *al,* 2018). Presence of DPRs was assessed with a quantitative and sensitive immunoassay for GP and GA and with a sensitive dot blot assay for GR and PR. Interestingly, this model also revealed antisense repeat RNA to induce toxicity independent of DPRs (Swinnen *et* *al,* 2018).

Overall, *Drosophila* models seem to be very sensitive to arginine‐rich DPR‐induced toxicity (Table 2). As such, the slightest presence of GR or PR generated through RAN translation in repeat expansion *Drosophila* models might mask potential RNA toxicity.

**Box 2. “RNA only constructs” and the search for the holy grail**

Expression constructs harboring the pure GGGGCC repeat sequences (i.e., “ATG repeat” and “non‐ATG repeat”—Fig 4) are easily confounded by the generation of RAN peptides and are therefore not well suited to assess RNA toxicity. Therefore, a GGGGCC repeat construct in which no RAN translation takes place would be the ideal paradigm. Mizielinska *et* *al* (2014) have generated “RNA only” constructs that lack an ATG start codon and contain the GGGGCC sequence which is regularly (every 15 repeats) interrupted by stop codons in all reading frames, both in the sense and in the antisense direction. As such, any toxicity arising from these constructs can only be attributed to RNA toxicity. “RNA only” constructs were initially found not to be toxic in *Drosophila* (Mizielinska *et* *al,* 2014), arguing against RNA toxicity. We as well as others, however, found that these constructs can induce (limited) neuronal toxicity in zebrafish and *Drosophila* (Moens *et* *al,* 2018; Swinnen *et* *al,* 2018). The limited toxicity could be explained in two ways. First, the “RNA only” repeat RNA might not resemble the physiological situation, as the original repeat sequence is regularly interrupted by non‐repeat sequences. This might disrupt the secondary structure of the repeat RNA and therefore interfere with its interaction with proteins. Second, the absence of toxicity with the “RNA only” constructs might be due to differences in the RNA‐binding protein pool in models *versus* humans, rendering the former more resistant to RNA toxicity. The observation that knockdown of the *Drosophila* orthologue of *HNRNPH* is harmless underscores this view (Moens *et* *al,* 2018), especially since *HNRNPH* knockdown is detrimental in human cells (Lefave *et* *al,* 2011). Altogether, “RNA only” constructs have so far not provided conclusive data on the role of RNA toxicity in disease pathogenesis.

#### Patient‐derived cellular disease models

Disentangling the pathological mechanisms at play in C9 patient‐derived disease models (Table 5) is complex and in addition to potential roles of RNA and DPR toxicity, loss of function also needs to be taken into consideration. Various reported phenotypes have been rescued by ASO‐mediated decrease in *C9ORF72* transcript levels (containing the repeat RNA), strongly arguing for a gain‐of‐function mechanism (Donnelly *et* *al,* 2013; Sareen *et* *al,* 2013; Zhang *et* *al,* 2015). Presence of DPRs has often not been assessed, and as discussed above, issues with detecting DPRs complicate the matter regarding the relationship between toxicity and DPRs in C9 cells. In general, DPRs are difficult to detect in C9 cells and have not been found in an aggregated state. Detection of antisense DPRs is even more challenging and PA has never been detected (Westergard *et* *al,* 2016). RNA foci, if assessed, were invariably present, hence any correlation with toxicity was absent. There is one study using patient‐derived iMNs supporting RNA toxicity (Donnelly *et* *al,* 2013). Treatment of C9 iMNs rescued the observed phenotype and reduced RNA foci but had no effect on GP expression (Donnelly *et* *al,* 2013). However, the presence of GR, PR, and GA was not assessed (Donnelly *et* *al,* 2013).

While a few *in vivo C9ORF72* models have provided some support for a role of RNA toxicity in these models, more work is needed to support this and to dissect DPR from RNA toxicity. When modeling repeat RNA toxicity, the original GGGGCC repeat sequence needs to be used. Therefore, these models have an unavoidable propensity to generate DPRs through RAN translation, making it almost impossible to discriminate RNA toxicity from DPR toxicity.

### Mislocalization of repeat RNA‐binding proteins (rRBPs)

In the second step of the pathological cascade of RNA toxicity, the repeat RNA interacts with several RBPs (Fig 3). Demonstrating this interaction in a disease‐relevant context is very challenging. rRBP mislocalization is generally used as a surrogate marker to indicate this interaction, hence providing indirect support for RNA toxicity. This mislocalization consists of a colocalization with RNA foci and/or a subcellular mislocalization. An overview of the involvement of rRBPs is given in Table 6.

**Table 6:**
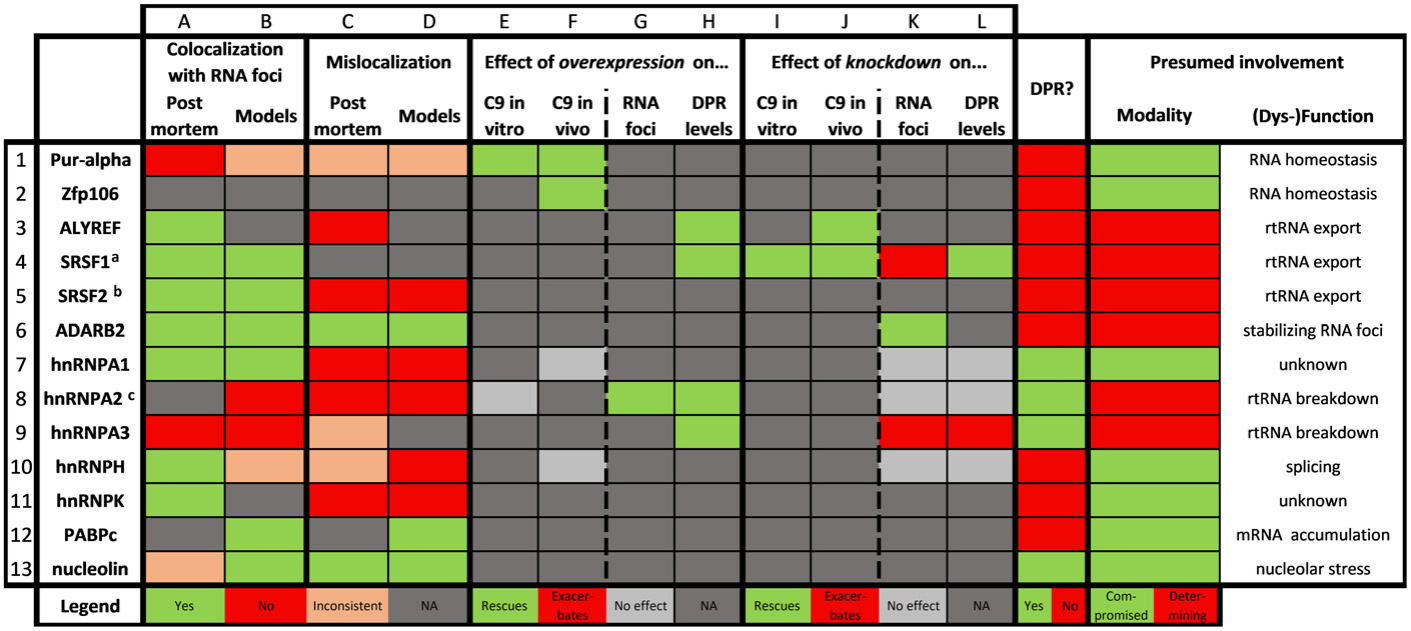
Mechanistic involvement of repeat RNA‐binding proteins in C9ORF72 ALS/FTD

For a subset of RNA‐binding proteins known to bind the C9ORF72 hexanucleotide repeat RNA, their individual involvement in C9ORF72 RNA toxicity is reviewed systematically. Subcellular mislocalization and colocalization with RNA foci in disease models as well as *post‐mortem* are depicted. The effect of overexpression and/or knockdown of the protein in C9 disease models is reviewed at four levels; effect on the toxicity in *in vitro* models, effect on the toxicity in *in vivo* models, effect on RNA foci, effect on DPR levels. For each protein, their possible involvement in DPR toxicity is depicted as well. Finally, based on the current literature, the presumed modality (compromised vs determining) and mechanism of their involvement are listed. Color legends are indicated at the bottom of the table. Abbreviations: NA, not assessed; rtRNA, repeat RNA.

References: 1A (Lee *et* *al,* 2013b); 1B (Sareen *et* *al,* 2013; O'Rourke *et* *al,* 2015; Rossi *et* *al,* 2015); 1C, (Lee *et* *al,* 2013b; Xu *et* *al,* 2013); 1D (Donnelly *et* *al,* 2013; Xu *et* *al,* 2013; Rossi *et* *al,* 2015); 1E (Xu *et* *al,* 2013); 1F (Xu *et* *al,* 2013; Swinnen *et* *al,* 2018); 2F (Celona *et* *al,* 2017); 3A (Cooper‐Knock *et* *al,* 2014, 2015b); 3C (Cooper‐Knock *et* *al,* 2015b); 3H (Hautbergue *et* *al,* 2017); 3J, (Freibaum *et* *al,* 2015; Hautbergue *et* *al,* 2017); 4A (Lee *et* *al,* 2013b); 4B (Lee *et* *al,* 2013b; Stopford *et* *al,* 2017); 4H (Hautbergue *et* *al,* 2017); 4I (Hautbergue *et* *al,* 2017); 4J (Hautbergue *et* *al,* 2017); 4K (Hautbergue *et* *al,* 2017); 4L (Hautbergue *et* *al,* 2017); 5A (Lee *et* *al,* 2013b; Cooper‐Knock *et* *al,* 2014, 2015b); 5B (Lee *et* *al,* 2013b; Stopford *et* *al,* 2017); 5C (Cooper‐Knock *et* *al,* 2015b); 5D (Yin *et* *al,* 2017); 6A (Donnelly *et* *al,* 2013); 6B (Donnelly *et* *al,* 2013); 6C (Donnelly *et* *al,* 2013); 6D (Donnelly *et* *al,* 2013); 6K, (Donnelly *et* *al,* 2013); 7A (Cooper‐Knock *et* *al,* 2014, 2015b); 7B (Sareen *et* *al,* 2013); 7C (Cooper‐Knock *et* *al,* 2015b; Fifita *et* *al,* 2017); 7D (Donnelly *et* *al,* 2013; Yin *et* *al,* 2017); 7F (Swinnen *et* *al,* 2018); 7K (Mori *et* *al,* 2016); 7L (Mori *et* *al,* 2016); 8B (Almeida *et* *al,* 2013; Sareen *et* *al,* 2013; O'Rourke *et* *al,* 2015); 8C (Fifita *et* *al,* 2017); 8D (Almeida *et* *al,* 2013); 8E (Xu *et* *al,* 2013); 8G (Mori *et* *al,* 2016); 8H (Mori *et* *al,* 2016); 8K (Mori *et* *al,* 2016); 8L (Mori *et* *al,* 2016); 9A (Lee *et* *al,* 2013b); 9B (Sareen *et* *al,* 2013; O'Rourke *et* *al,* 2015); 9C (Lee *et* *al,* 2013b; Mori *et* *al,* 2013b; Boeynaems *et* *al,* 2016; Davidson *et* *al,* 2017; Fifita *et* *al,* 2017); 9H (Mori *et* *al,* 2016); 9K (Mori *et* *al,* 2016); 9L (Mori *et* *al,* 2016); 10A (Lee *et* *al,* 2013b; Cooper‐Knock *et* *al,* 2014, 2015b); 10B (Almeida *et* *al,* 2013; Lee *et* *al,* 2013b; O'Rourke *et* *al,* 2015; Rossi *et* *al,* 2015; Conlon *et* *al,* 2016); 10C (Cooper‐Knock *et* *al,* 2015b; Conlon *et* *al,* 2016); 10D (Almeida *et* *al,* 2013); 10F (Swinnen *et* *al,* 2018); 10K (Mori *et* *al,* 2016); 10L (Mori *et* *al,* 2016); 11A (Cooper‐Knock *et* *al,* 2015b); 11C (Cooper‐Knock *et* *al,* 2015b); 11D (Haeusler *et* *al,* 2014); 12B (Rossi *et* *al,* 2015); 12D (Rossi *et* *al,* 2015); 13A (Haeusler *et* *al,* 2014; Cooper‐Knock *et* *al,* 2015b; Stopford *et* *al,* 2017); 13B (Stopford *et* *al,* 2017); 13C (Cooper‐Knock *et* *al,* 2015b); 13D (Haeusler *et* *al,* 2014)^,^(O'Rourke *et* *al,* 2015).

a.k.a. SF1.

a.k.a. SC35.

a.k.a. hnRNPA2/B1.

Mislocalization of rRBPs has been demonstrated in some patient‐derived *in vitro* models. Most importantly, *post‐mortem* examination revealed mislocalization of numerous rRBPs. Sense nuclear RNA foci have been shown to contain SRSF1, SRSF2, ALYREF, ADARB2, HNRNPA1, HNRNPH, and HNRNPF (Donnelly *et* *al,* 2013; Lee *et* *al,* 2013a; Cooper‐Knock *et* *al,* 2014). Antisense nuclear RNA foci have been demonstrated to colocalize with SRSF2, ALYREF, HNRNPA1, HNRNPH, and HNRNPK (Cooper‐Knock *et* *al,* 2015b). However, some potential limitations of these studies should be mentioned. The colocalization was often not demonstrated in disease‐relevant tissue (i.e., frontal cortex, motor cortex, and spinal cord). Moreover, colocalization data are often conflicting between different studies, probably related to methodological differences. Finally, some DPRs (i.e., GR and PR) also interact and/or colocalize with hnRNPs (Table 6). Therefore, the mislocalization and/or dysfunction of these rRBPs might be related to DPR toxicity.

Importantly, one needs to keep in mind that the absence of rRBP mislocalization or colocalization with RNA foci does not exclude mislocalization as the sensitivity of conventional immunohistochemistry might be too low to detect a (subtle) altered subcellular distribution. This makes validation of rRBP mislocalization in *post‐mortem* tissue very challenging.

### Signatures of rRBP dysfunction

In the third step of the pathological cascade of RNA toxicity, the function of several rRBPs is compromised. Monitoring signatures of this dysfunction might be an indirect proof of RNA toxicity. Given the complexity of this pleiotropic pool of rRBPs, only a limited amount of rRBP dysfunction hallmarks have been identified so far. An increase in splicing errors has been noted in *C9ORF72* iMNs (Cooper‐Knock *et* *al,* 2015a), which was confirmed in C9 ALS/FTD brains and mainly constituted intron retention events (Prudencio *et* *al,* 2015). This is in line with several rRBPs having a cardinal role in RNA splicing. Moreover, a large proportion of the misspliced transcripts in C9 ALS/FTD were targets of HNRNPH and SRSF1, indicating their dysfunction (Prudencio *et* *al,* 2015; Conlon *et* *al,* 2016). Additionally, transcriptomics in patient‐derived material generally indicates an alteration of genes involved in RNA metabolism thereby circumstantially suggesting rRBP misregulation (Chew *et* *al,* 2015; Cooper‐Knock *et* *al,* 2015a; Selvaraj *et* *al,* 2018). More research will be needed to investigate downstream signatures of rRBP dysfunction. Given the large functional pleiotropy of each of these rRBPs, identifying these disturbances will be challenging.

### Clinicopathological correlations

In the final step of the pathological cascade of RNA toxicity, neurodegeneration results in clinical phenotypic manifestations. Demonstrating a correlation between key clinical or pathological features and hallmarks of RNA toxicity would be the best proof of RNA toxicity. Indeed, the burden of sense RNA foci was inversely correlated with age at onset in C9 FTD patients (Mizielinska *et* *al,* 2013). Moreover, the burden of sense RNA foci in spinal motor neurons was higher in C9 ALS than in C9 FTD patients, suggesting RNA foci to be correlated with the clinical phenotype (Cooper‐Knock *et* *al,* 2014). In addition, the splicing error rate in C9 iMNs and lymphoblastoids was correlated with disease severity (Cooper‐Knock *et* *al,* 2015a). Despite these correlations, most clinical and pathological data do not provide a correlation with RNA toxicity hallmarks. Several studies identified no detrimental associations between RNA foci and clinicopathological features (Gendron *et* *al,* 2013; Mizielinska *et* *al,* 2013; DeJesus‐Hernandez *et* *al,* 2017). On the contrary, a higher antisense RNA foci burden was even correlated with a delayed age at onset (DeJesus‐Hernandez *et* *al,* 2017). Moreover, RNA foci generally do not follow the pattern of neurodegeneration and TDP‐43 pathology (Gendron *et* *al,* 2013; Mizielinska *et* *al,* 2013; DeJesus‐Hernandez *et* *al,* 2017). Finally, there has been no correlation between RNA foci and toxicity in any of the repeat expansion models developed so far, both *in vitro* and *in vivo* (Tables 3, 4, 5).

## Mechanisms of RNA toxicity: new insights from C9 ALS/FTD

### rRBP loss of function

Using pulldown approaches, the interacting partners of repeat RNA have been studied extensively (Table 7). Classical RNA‐binding proteins (RBPs) constitute the majority of the interactome. These mainly include hnRNPs and mRNA transport proteins. Notably, there is a large variability in the identified proteins between studies likely due to methodological differences (e.g., cell lysate species, probe length, and/or identification method). Additionally, the repeat length was below the presumed threshold of toxicity in most studies (i.e., ± 30 repeats). While it is clear that *C9ORF72* repeat RNA interacts with a large number of proteins, the involvement of RNA toxicity as well as its pathological underpinnings is still unclear.

**Table 7:**
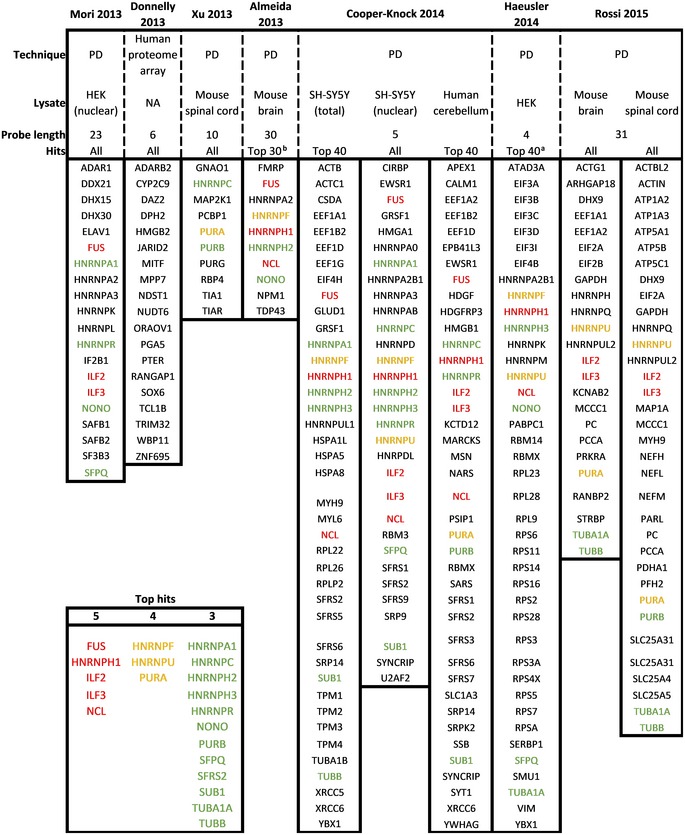
Sense repeat RNA interactome

Overview of proteins identified to bind sense (GGGGCC) repeat RNA. In case the list of identified proteins exceeded 40 hits, only the top 40 proteins were included. Box inset left bottom indicates most frequently identified (five times = red, four times = orange, three times = green) proteins.

*Abbreviations*: NA, not available; PD, pulldown.

Based on “unique peptides”.

Incomplete list in original manuscript.

Research on some of the rRBPs in several *C9ORF72* models has corroborated the idea of repeat RNA interfering with their normal function, leading to a loss of function of the rRBP (Table 6). First, overexpression of some of these rRBPs rescued the phenotype induced by the repeat expansion [i.e., Pur‐alpha (Xu *et* *al,* 2013; Swinnen *et* *al,* 2018) and Zfp106 (Celona *et* *al,* 2017)], indicating that repeat RNA might impair their functionality and/or protein level. Additionally, knockdown of these rRBPs in wild‐type models was detrimental (Xu *et* *al,* 2013; Celona *et* *al,* 2017), suggesting that dysfunction of these rRBPs is harmful. Second, for some rRBPs [Pur‐alpha (Rossi *et* *al,* 2015), ADARB2 (Donnelly *et* *al,* 2013), nucleolin (Cooper‐Knock *et* *al,* 2015b), hnRNPA3 (Boeynaems *et* *al,* 2016), and hnRNPH (Conlon *et* *al,* 2016)], the repeat expansion altered their subcellular distribution. This indicates that the physical interaction between repeat RNA and rRBPs might lead to a functional sequestration of the latter.

When evaluating the repeat RNA interactome (Table 7), FUS is one of the proteins most consistently shown to bind to repeat RNA. This suggests that FUS dysfunction could contribute to C9 ALS/FTD pathogenesis and hence hints toward convergent mechanisms between *FUS* ALS and *C9ORF72* ALS. In addition, nucleolin is shown in several studies to bind C9 ALS/FTD repeat RNA, suggesting that repeat RNA could directly induce nucleolar stress. This might constitute a convergence point between RNA toxicity and DPR toxicity as DPRs are believed to lead to nucleolar stress (Balendra & Isaacs, 2018). The consistently observed interaction of repeat RNA with ILF2 and ILF3 might also mediate various dysregulations of RNA metabolism. These proteins are known to bind DNA:RNA hybrids and are involved in dynamics of various RNA granules as well as in nucleolar homeostasis and splicing (Shiina & Nakayama, 2014; Nadel *et* *al,* 2015; Wandrey *et* *al,* 2015). Surprisingly, albeit less consistent, repeat RNA has regularly been reported to bind cytoskeletal proteins (e.g., TUBB and TUBA1A), possibly contributing to axonal dysfunction of motor neurons like has been described for FUS mutations (Guo *et* *al,* 2017).

**Figure 5:**
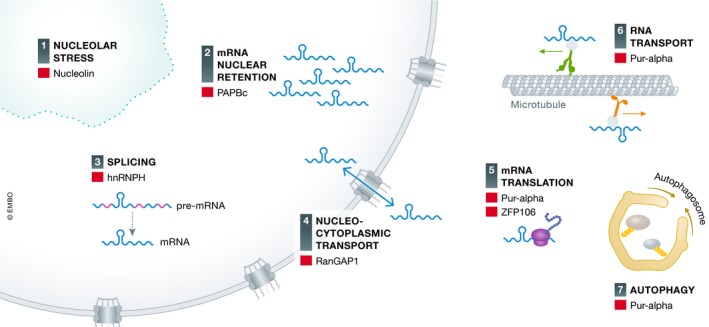
Processes possibly disturbed by C9ORF72 RNA toxicity (1) Compromised function of nucleolin (NCL) might induce nucleolar stress. (2) mRNA might be retained in the nucleus due to repeat RNA‐induced nuclear accumulation of mRNA export proteins like PABPC. (3) Splicing might be disturbed due to compromised function of several splicing factors like HNRNPH. (4) Nucleocytoplasmic transport might be directly disturbed by repeat RNA via RanGAP1 dysfunction. (5) Translation of mRNA might be altered due to compromised function of translational factors like Pur‐alpha and ZFP106. (6) Cytoplasmic RNA transport might be disturbed by compromised function of RNA transport factors like Pur‐alpha. (7) Autophagy might be compromised by dysfunction of Pur‐alpha.

The presumed loss‐of‐function of several rRBPs might lead to disturbances in several cellular processes (Fig 5). Given that many rRBP processes are related to RNA metabolism like splicing (e.g., HNRNPH), nuclear mRNA export (e.g., PABPC), mRNA translation (e.g., ZFP106), cytoplasmic RNA transport (e.g., PURA), and nucleolar stress (e.g., nucleolin), this could lead to cellular stress via different pathways. However, the exact contribution and net effect of each rRBP in RNA toxicity is a puzzle that still needs to be solved.

### rRBPs contribute to repeat RNA localization dynamics

As discussed above, repeat RNA has been shown to induce dysfunction of several rRBPs. Moreover, repeat RNA has also been shown to affect localization of a subset of rRBPs (Table 6). This is illustrated by the intriguing observation that some rRBPs alleviate the repeat toxicity upon knockdown instead of overexpression. Several of these rRBPs are involved in mRNA transport including ALYREF (Hautbergue *et* *al,* 2017), SRSF1 (Hautbergue *et* *al,* 2017), and FMRP (Burguete *et* *al,* 2015). This implies that rRBPs might regulate the subcellular localization of repeat RNA and indirectly contribute to RNA (and DPR) toxicity. Indeed, three independent findings support this interpretation. First, SRSF1 knockdown increased nuclear and decreased cytoplasmic RNA foci (Hautbergue *et* *al,* 2017), suggesting that SRSF1 mediates the nuclear export of repeat RNA to the cytoplasm. Similar findings were obtained for ALYREF, another known mRNA export protein (Hautbergue *et* *al,* 2017). Second, ADARB2 knockdown reduced the amount of nuclear RNA foci (Donnelly *et* *al,* 2013), indicating that it might be involved in the nuclear retention of repeat RNA or that it might have a stabilizing effect on RNA foci. Third, FMRP knockdown decreased the toxicity induced by repeat RNA located in the neurites (Burguete *et* *al,* 2015), while it also colocalized with this repeat RNA suggesting that FMRP might be responsible for the transport of repeat RNA to the neurites. These studies support that a subset of rRBPs have the ability to regulate the toxic potential of the repeat RNA by affecting its stability and/or subcellular transport.

### Nuclear versus cytoplasmic repeat RNA

In line with the observation that RNA transport factors modify repeat RNA toxicity, the localization of the repeat RNA seems to be crucial for its toxicity. It is still unclear whether the nuclear or the cytoplasmic repeat RNA species are the most toxic ones or whether they both contribute to toxicity. Analysis in *post‐mortem* samples shows that nuclear RNA foci are more abundant than cytoplasmic ones. However, since nuclear RNA foci show very little to no correlation with neurodegeneration, their pathogenic role is unclear. Increasing evidence supports the notion of cytoplasmic repeat RNA being the main culprit. Cytoplasmic RNA foci have been detected in *post‐mortem* tissue (Cooper‐Knock *et* *al,* 2015b), and sense repeat RNA was found in neurites of C9 iMNs (Burguete *et* *al,* 2015). Moreover, the existence of cytoplasmic repeat RNA is a prerequisite for DPR production. As a consequence, the presence of DPRs could be considered as an indirect proof of cytoplasmic repeat RNA. Additionally, decrease of cytoplasmic RNA foci by SRSF1 knockdown was beneficial (Hautbergue *et* *al,* 2017). Finally, repeat RNA localized in the neurites was sufficient to cause toxicity (Burguete *et* *al,* 2015).

The hypothesis that cytoplasmic repeat RNA is the main culprit still leaves the discussion open between DPR and RNA toxicity, as DPRs are generated from cytoplasmic repeat RNA. Moreover, it is still unclear in which transcriptional context the repeat RNA is generated (Fig 1). In case it is mainly generated from intron 1 retaining transcripts, the repeat RNA should indeed have a high propensity for cytoplasmic localization as it has a polyA tail. In contrast, if it is mainly generated from spliced‐out intron 1 or abortive transcripts, the repeat RNA is more likely to be retained in the nucleus.

### RNA foci versus soluble repeat RNA

While RNA foci are an important hallmark of C9 ALS/FTD, their involvement in disease pathogenesis is still elusive. In fact, current *post‐mortem* data do not support RNA foci as the driver of neurodegeneration. First, similar to DPRs, they do not follow the pattern of neurodegeneration, as RNA foci are equally present in non‐affected regions (e.g., cerebellum and hippocampus) with the highest level in cerebellar Purkinje cells (Mackenzie *et* *al,* 2014; Saberi *et* *al,* 2015; DeJesus‐Hernandez *et* *al,* 2017). Second, the presence of RNA foci does not correlate with TDP‐43 pathology (Mizielinska *et* *al,* 2013), even though some correlation in motor neurons between antisense RNA foci and TDP‐43 pathology has been suggested (Cooper‐Knock *et* *al,* 2015b). Third, an extensive study identified no detrimental associations between RNA foci and clinical features (DeJesus‐Hernandez *et* *al,* 2017). On the contrary, a higher antisense burden of RNA foci was correlated with a delayed age at onset (DeJesus‐Hernandez *et* *al,* 2017). Current *in vitro* and *in vivo* data further question the disease relevance of RNA foci. Most importantly, neither toxicity nor TDP‐43 pathology is correlated with the presence of RNA foci, both in non‐ATG GGGGCC models (Tables 3 and 4) and in patient‐derived *in vitro* models (Table 5). Altogether, this evidence seems to be in line with the idea that RNA foci do not contribute significantly to C9 ALS/FTD pathogenesis. As a consequence, repeat RNA not confined in RNA foci (i.e., “soluble repeat RNA”) could be the real perpetrator of RNA toxicity. However, assessing soluble repeat RNA is very challenging and so far only one study has been able to visualize soluble repeat RNA species (Burguete *et* *al,* 2015).

### Lessons from other non‐coding repeat expansion disorders for RNA toxicity

While the mechanism and exact contribution of RNA toxicity in the other non‐coding repeat expansion disorders is still poorly understood, they can provide important insights into the existence and mechanism of RNA toxicity (in C9 ALS/FTD).

First, analysis in other non‐coding repeat expansion disorders questions the pathogenic role of nuclear RNA foci. Instead of being stable aggregates of repeat RNA sequestering RNA‐binding proteins, work in myotonic dystrophy type 1 supports the notion that RNA foci are highly dynamic structures, with several RNA‐binding proteins themselves being involved in this dynamic process (Lopez‐Morato *et* *al,* 2018). As a consequence, nuclear RNA foci might not entirely account for rRBP dysfunction suggesting another mediator of toxicity. Interestingly, cytoplasmic soluble repeat RNA seems a possible candidate. Also in myotonic dystrophy type 1, repeat RNA has been demonstrated to reside in the cytoplasm as well, often adapting a single mRNP conformation (i.e., soluble repeat RNA), opposed to RNA foci (Pettersson *et* *al,* 2015). Interestingly, work in FXTAS has shown that the function of several rRBPs (e.g., Pur‐alpha and hnRNPA2) can be altered without them physically being mislocalized (Boivin *et* *al,* 2018). Therefore, rRBP mislocalization cannot necessarily always be equated with rRBP dysfunction.

Second, several rRBPs that are able to rescue in models of these diseases have also been implicated in C9 ALS/FTD, suggesting mechanistic commonalities. These include Pur‐alpha (FXTAS; Boivin *et* *al,* 2018), hnRNPA2 [FXTAS (Boivin *et* *al,* 2018), and SCA31 (Ishiguro *et* *al,* 2017)] as well as hnRNPK (SCA10; White *et* *al,* 2010). Interestingly, despite commonalities, the pool of involved rRBPs (Table 1) is considerably divergent between different diseases. This might partially underlie the observed pathological and clinical differences.

Third, the repeat sequence in SCA36 (TGGGCC) is strikingly similar to the GGGGCC repeat expansion in C9 ALS/FTD. Moreover, SCA36 and C9 ALS/FTD are the only non‐coding repeat expansion disorders with significant motor neuron involvement (Kobayashi *et* *al,* 2011; Ikeda *et* *al,* 2012), suggesting that a similar mechanism might be at play. Interestingly, two RAN proteins (GP and PR) are mutual as well as several rRBPs. This observation makes a loss‐of‐function unlikely to be the main mediator of toxicity. Moreover, it indicates that DPR toxicity is insufficient to explain the pathogenesis, as GP has no toxic potential (as described previously) and as PR is only sporadically detected, suggesting an important role for RNA toxicity.

## How to disentangle RNA from RAN—Future directions

Deciphering the exact pathogenic code underlying each of the non‐coding repeat expansion disorders is important to identify therapies to halt these aggressive diseases. To maximize the effectiveness of therapies, the exact involvement of each of the three possible mechanisms (RNA, RAN and loss‐of‐function) needs to be uncovered. Assessing whether RNA toxicity, alone or in addition to RAN toxicity, plays a substantial pathogenic role is crucial to predict the effectiveness of RAN directed therapies (e.g., nanobodies). At this moment, a multimodal therapeutic strategy aiming at all three possible mechanisms (or at least the two gain‐of‐function mechanisms) has the best potential of clinical success.

### Disease models

As already pointed out, assessment of RNA toxicity in disease models is difficult since “non‐ATG repeat RNA constructs” have the intrinsic propensity to undergo RAN translation (cf. Box 1). Therefore, both repeat RNA and RAN proteins are present in most models, precluding a clean assessment of RNA toxicity. Unfortunately, even “RNA only constructs” are not optimal either (cf. Box 2). Different approaches to assess pure RNA toxicity, in a RAN‐devoid context, are highly needed. Possible paradigms include the use of specific RAN translation inhibitors or the induction of RAN peptide degradation. In this regard, further research into the exact mechanism underlying RAN translation will be crucial to develop these strategies. For the time being, an adequate assessment of RAN proteins in all models exploiting “non‐ATG repeat RNA constructs” is pivotal.

Another important issue is the need to assess all possible toxic repeat RNA species. Whereas research so far has been highly biased toward RNA foci, soluble repeat RNA has been given very little attention. Unfortunately, visualizing this soluble repeat RNA is very difficult. Approaches like MS2 tagging (Burguete *et* *al,* 2015) will need to be further refined and exploited.

Regarding rRBP dysfunction, for each non‐coding repeat expansion disorder the pool of rRBPs needs to be identified and validated, e.g., by performing additional pulldown approaches and bioinformatical modeling of the interactions. Moreover, the effect of its overexpression as well as knockdown needs to be determined for each identified rRBP in relevant disease models in order to assess its functional implication in disease pathogenesis. Similarly, rRBP subcellular localization as well as downstream disturbances needs to be investigated more extensively in disease models.

Altogether, disease models need to be exploited more efficiently. Ideally, each model should be completely and thoroughly characterized, and RAN peptide presence should be assessed systematically using an adequate methodology.

### Post‐mortem research

So far, most research has been performed in disease models. As *post‐mortem* research has an obviously higher disease relevance, it should be given much more attention. Brain and spinal cord tissue should be obtained more systematically and used judiciously. Similar to research in disease models, more attention should be given to soluble repeat RNA species. Moreover, rRBP dysfunction assessment should comprise more than just assessment of colocalization with RNA foci. Downstream signatures (e.g., splicing defects) of rRBP dysfunction should also be investigated (e.g., by RNA sequencing).

### Patient research

Research in living patients is of highest disease relevance. Imaging approaches aiming to visualize RAN proteins (e.g., through a PET (positron emission tomography) tracer) might provide valuable information and may be able to establish a temporal relationship with disease milestones. Also, fluid biomarkers (e.g., DPRs or repeat RNA in cerebrospinal fluid and/or blood) might be another approach to gauge the relation between these disease mechanisms and clinical aspects. These approaches should ideally be combined with prospective long‐term follow‐up studies of presymptomatic individuals carrying a *C9ORF72* repeat expansion.

## Conclusions

### Non‐coding repeat expansion disorders in general

Three possible mechanisms might underlie pathogenesis in non‐coding repeat expansion disorders; RNA toxicity, RAN toxicity, and loss of function. With the currently available information, we conclude that RNA toxicity might contribute to the disease mechanism in all diseases. However, it has not been assessed in SCA12 so far. RAN toxicity seems possible in all diseases except SCA12 as presence of RAN protein inclusions in *post‐mortem* material was positively excluded. Contribution of RAN toxicity in SCA10 and SCA36 is still largely elusive as the presence of RAN proteins in *post‐mortem* tissue has not reliably been established yet. Loss of function could eventually only play a significant pathogenic role in SCA12, where an “alteration‐of‐function” instead of a loss‐of‐function is suspected.

Data so far have been inconclusive in determining the exact contribution of RNA toxicity, RAN toxicity, and loss of function in the pathogenesis of non‐coding repeat expansion disorders. As all non‐coding repeat expansion disorders probably share a similar underlying pathogenesis, a holistic research approach concerning these disorders needs to be implemented. Pathogenic differences and commonalities between these disorders are the key to unravel the exact contribution of RNA toxicity, RAN toxicity, and loss‐of‐function. Of interest, several rRBPs are shared between different repeat RNA interactomes, with MBNL1, CUGBP1, PURA, HNRNPA2, HNRNPK, SRSF1, and SRSF2 being shared by at least two disorders.

### C9 ALS/FTD

While C9 ALS/FTD mainly seems to be a gain‐of‐function disease, it is currently unclear whether this is mediated by DPR and/or RNA toxicity. Given the toxicity in several *in vitro* and *in vivo* models, DPRs could be involved. While DPRs have been modeled extensively, research into RNA toxicity is still limited, mainly due to technical challenges in modeling RNA toxicity. Despite these methodological problems, we conclude that evidence favoring the existence of RNA toxicity is increasing and that this toxicity could induce alterations in splicing, nucleolar function, mRNA nuclear export, cytoplasmic RNA transport, and autophagy. Further research to establish the exact role of RNA toxicity in C9 ALS/FTD needs to be structured along the pathogenic cascade of repeat RNA toxicity. Such research will be crucial in guiding clinical research to develop new therapeutic approaches for C9 ALS/FTD.

## Author contributions

BS performed the literature search and wrote the manuscript. LVDB and WR discussed the literature and co‐wrote the manuscript.

## Conflict of interest

The authors declare that they have no conflict of interest.
